# Ultrasound evaluation of patients with male accessory gland inflammation: a pictorial review

**DOI:** 10.1111/andr.13011

**Published:** 2021-05-04

**Authors:** Sandro La Vignera, Andrea Crafa, Rosita A. Condorelli, Federica Barbagallo, Laura M. Mongioì, Rossella Cannarella, Michele Compagnone, Antonio Aversa, Aldo E. Calogero

**Affiliations:** ^1^ Department of Clinical and Experimental Medicine University of Catania Catania Italy; ^2^ Department of Experimental and Clinical Medicine Magna Graecia University of Catanzaro Catanzaro Italy

**Keywords:** male accessory gland infections, prostate and vesicular ultrasound, scrotal ultrasound, integrated Stamey test

## Abstract

Male accessory gland infection/inflammation (MAGI) is a major cause of male infertility. The importance of ultrasound evaluation in these patients is highly controversial, although evidence of its relevance has increased in recent years. Ultrasound criteria are an important element for confirming the clinical diagnosis. Furthermore, they help to localize the anatomical site or sites of the inflammatory process and in assessing its extension which, in turn, have different consequences on the seminological and symptomatic aspects. This article summarizes the clinical interpretations related to ultrasound aspects in patients with MAGI and the possible effects on the seminological, microbiological, endocrinologic, urological, sexological, and internist aspects.

## 1 INTRODUCTION

Male accessory gland infection/inflammation (MAGI) represents a nosographic category responsible for male infertility^1^ with a prevalence ranging from 2% to 18% of infertile patients.^2^ Indeed, according to the World Health Organization (WHO) criteria, MAGI can be established when a patient has oligo‐, astheno‐, and/or teratozoospermia associated with at least one factor A (history of genitourinary infection or physical signs) plus one factor B (abnormality of prostatic fluid), one factor A plus one factor C (ejaculate signs), one factor B plus one factor C, or two factors C.^3^

Several mechanisms may contribute to the alteration of sperm parameters in patients with MAGI. These include the obstruction of the ejaculatory ducts, oxidative stress and imbalance of cytokines, impaired secretory capacity of the sex glands, and direct microbial damage. In this contest, ultrasound (US) characterization of patients with MAGI is routinely performed by evaluating prostate, seminal vesicles, and epididymis. However, in clinical practice the usefulness of US is controversial. Indeed, although their use is widespread, the specificity and sensitivity of this diagnostic tool are not considered very high for these specific conditions.^4^

The American Institute of Ultrasound in Medicine (AIUM) suggests using transrectal ultrasound (TRUS) to evaluate the prostate and the seminal vesicles in all infertile patients. Other indications are represented by (a) echo‐guided biopsy for prostate nodules suspected of malignancy by digital rectal examination or for elevated serum prostate‐specific antigen (PSA) levels, and/or magnetic resonance imaging suggestive of prostate malignancy; (b) calculation of the prostate volume before surgical procedures and/or radiotherapy, and for the calculation of PSA density; (c) a guide for positioning the needles for radiotherapy; (d) evaluation of functional disorders associated with lower urinary tract symptoms (LUTS); (e) to study of morphostructural congenital anomalies; (f) hemospermia; (g) disease recurrence in patients with previous prostatectomy.^5^ The European Association of Urology (EAU) guidelines suggest the use of TRUS only for patients with a suspicion of obstructive azoospermia and, in particular, when the lack of spermatozoa in the ejaculate is associated with a low volume.

US evaluation of the epididymal tract should be limited to patients with signs indicative of obstruction. These include dilation of the rete testis, cystic dilation of the cephalic tract, suspected absence of the vas deferens.^1^ In particular, Pezzella and colleagues showed that when the longitudinal diameter of the epididymal cephalic tract is >0.85 mm associated with serum FSH levels <7.8 IU/ml, it is suggestive of obstructive azoospermia (sensitivity 58.8%; specificity 91.4%).^6^ A recent study conducted by the European Academy of Andrology (EAA) confirmed that the dilation of the head and tail of the epididymis is associated with the presence of acute and chronic inflammation, distal obstruction, and with a positive MAR test, suggesting an association of this finding with alteration of the blood‐epididymal barrier.^7^ A systematic review has shown that the TRUS evaluation of epididymal and of the prostate‐vesicular region is useful in the clinical evaluation of patients with chronic inflammation of these anatomical sites suggesting the use of TRUS in the characterization of MAGI.^8^ Moreover, a US scan allows more accurate classification of MAGI by identifying the number of glands involved in the inflammatory process as well as its extension. Accordingly, MAGI can be classified in uncomplicated (prostatitis alone) and complicated (prostate‐vesiculitis and prostate‐vesiculo‐epididymitis), and into unilateral or bilateral forms. This classification correlates with the outcome of fertility since complicated and bilateral forms have a worse impact on sperm parameters.^2^

Previously, we have reported US criteria suggestive for prostatitis, prostate‐vesiculitis), PVE (prostate‐vesiculo‐epididymitis) (Table 1).^9^ In particular, the diagnostic sensitivity and specificity of US examination increases as the number of US signs found increases. In fact, we have shown that the presence of more than two criteria of prostatitis is associated with a diagnostic sensitivity of 0.90 [confidence interval (CI) 95% = 0.78–0.97], specificity of 1.00 (CI 95% = 0.96–1.00), positive predictive value of 1.00 (CI 95% = 0.92–1.00), and negative predictive value of 1.00 (CI 95% = 0.89–0.98). Similarly, the presence of more than two ultrasound signs of vesiculitis has a sensitivity of 1.00 (CI 95% = 0.93–1.00), specificity of 0.98 (CI 95% = 0.93–0.99), positive predictive value of 0.96 (CI 95% = 0.86–0.99), and negative predictive value of 1.00 (CI 95% = 0.96–1.00). Finally, the presence of more than two signs of epididymitis was associated with a diagnostic sensitivity of 1.00 (CI 95% = 0.93–1.00), specificity of 1.00 (CI 95% = 0.96–1.00), positive predictive value of 1.00 (CI 95% = 0.93–1.00), and negative predictive value of 1.00 (CI 95% = 0.96–1.00).^10^ Furthermore, according to these criteria, we also identify two different US forms of complicated MAGI, the hypertrophic‐congestive (HCUF), and the fibro‐sclerotic form (FSUF), which differ not only for US aspect but also in the sperm outcome. Indeed, FSUF is associated with lower sperm motility, total number, and percentage of normally shaped spermatozoa than HCUF one. This latter has higher semen leukocytes concentration and seminal reactive oxygen species. Probably this difference is due to the fact that FSUF correlates with a chronic inflammatory process, while HCUF correlates with an acute process ^11^ (Table 2).

**TABLE 1:** Ultrasound criteria of MAGI^9^

Prostatitis is suspected in the presence of >2 of the following ultrasonographic signs:
asymmetry of the gland volume; areas of low echogenicity; areas of high echogenicity; dilatation of periprostatic venous plexus; single or multiple areas of acinar ectasia; area/s of moderate increased of vascularity (focal or multiple).
Vesciculitis is suspected in the presence of >2 of the following ultrasonographic signs:
increase (>14 mm) anteroposterior diameter (mono or bilateral); asymmetry >2.5 mm compared to the controlateral vesicle; reduced (<7 mm) anteroposterior diameter (mono or bilateral); glandular epithelium thickened and/or calcified; polycyclic areas separated by hyperechoic septa in one or both vesicles; fundus/body ratio >2.5 or fundus/body ratio <1; anteroposterior diameter unchanged after ejaculation.
Epididymitis is suspected in the presence of >2 of the following ultrasonographic signs:
increase in size of the head (cranio‐caudal diameter >12 mm) and/or of the tail (cranio‐caudal diameter >6 mm) (mono or bilateral); presence of multiple microcystis in the head and/or tail (mono or bilateral); low echogenicity or high echogenicity (mono or bilateral); large hydrocele mono or bilateral; enlargement in superior part of the cephalic tract and superior/inferior part ratio >1; unchanged anteroposterior diameter of tail after ejaculation.

**TABLE 2:** Ultrasound differences between the hypertrophic‐congestive (HCUF) and a fibro‐sclerotic (FSUF) form of MAGI^11^

Hypertrophic‐congestive ultrasound form (HCUF): simultaneous presence of the following US criteria
prostate: increase of volume, areas of ipoechogenicity, dilatation of periprostatic venous plexus, single or multiple internal similar cystis areas; seminal vesicles: increase of volume, mono‐ or bilateral increased (>14 mm) APD, polycyclic areas separated by hyperechoic septa in both vesicles, fundus/body ratio >2.5; epididymis: increased (>6 mm) tail cranio‐caudal diameter, bilateral head and tail areas of ipoechogenicity, unchanged tail APD just after ejaculation
Fibro‐sclerotic ultrasound form (FSUF): simultaneous presence of following US criteria:
prostate: areas of hyperechogenicity, asymmetry of the gland volume; seminal vesicles: reduced (<7 mm) mono‐ or bilateral APD, thickened and/or calcified glandular epithelium, fundus/body ratio <1; epididymis: bilateral head and tail areas of hyperechogenicity

## 2 LABORATORY ASPECT

The inflammatory process that occurs in MAGI correlates with the production of various cytokines that promote the production of oxygen free radicals (ROS), perpetuating the inflammatory process and thus further increasing sperm damage. Interleukin‐6 (IL‐6), interleukin‐8 (IL‐8), tumor‐necrosis factor‐α (TNFα), and interleukin‐1β (IL1β) are among the main cytokines that correlate with leukocytospermia and chronic prostatitis or chronic pelvic pain syndrome (CP/CPPS). Accordingly, these cytokines have been proposed as markers of MAGI.^12^ Some studies have shown how the levels of these cytokines not only correlate with the alteration of seminal parameters but also with US patterns. Indeed, we have previously highlighted that patients with US criteria of prostate‐vesicular‐epididymitis have seminal fluid concentrations of the anti‐inflammatory cytokine IL‐10 significantly lower and IL‐6 and TNFα concentrations significantly higher compared to patients with US criteria of prostate‐vesiculitis or prostatitis.^13^ In another study, conducted on 250 infertile patients, 79 of whom meet the criteria for MAGI, Lotti and colleagues found that IL‐8 levels correlated with tail epididymal alterations, such as hypo‐ or hyperechogenicity, presence of calcifications, hyperemia, and increased size. Moreover, IL‐8 showed a relationship with US prostate abnormalities, such as calcifications (in particular macrocalcifications >3 mm), inhomogeneous/hypoechoic texture, hyperemia, and high arterial blood flow. Finally, IL‐8 levels correlated with pre‐ejaculatory hyperechoic seminal vesicles.^14^

Patients with prostate‐vesicular‐epididymitis have lower seminal concentrations of fructose and significantly increased levels of ROS of leukocyte origin compared with patients with prostatitis alone. However, the seminal concentrations of fructose and ROS do not differ between patients with mono or bilateral inflammatory (amicrobic) prostate‐vesicular‐epididymitis, confirming the importance of US evaluation.^15^ The harmful effects of the inflammatory process that occurs in patients with MAGI on sperm quality are present not only on conventional sperm parameters but also on biofunctional ones. In fact, we have shown that 150 patients with MAGI have not only lower seminal fluid volume, sperm concentration, total sperm count, percentage of spermatozoa with normal forms, and progressive motility compared with controls, but also had a higher percentage of spermatozoa with low mitochondrial membrane potential (MMP), phosphatidyl‐serine (PS) externalization (a marker of apoptosis), and sperm DNA fragmentation (SDF), and a decreased percentage of alive spermatozoa (evaluated by annexin V/PI assay) than controls, without significant differences between patients with inflammatory or microbic form.^16^ Lotti and colleagues highlighted the ability of US to predict increased SDF in two different cytometry sperm populations of patients with infertility, thus strengthening the role of the US in understanding when the damage to the sperm component occurs. In greater detail, spermatozoa dimmer to the propidium iodide (PI) staining are non‐viable sperm and show signs of apoptosis, suggesting impaired testicular spermatogenesis. Accordingly, patients with PI^dimmer^ SDF show US signs suggestive of testicular abnormalities, such as low testicular volume, testicular inhomogeneity or hypoechogenicity, or epididymal tail inhomogeneity. Instead, spermatozoa brighter to the PI staining are the sum of viable and non‐viable spermatozoa, highly oxidized. The increased SDF of PI^brighter^ spermatozoa correlates with the presence of US prostate signs of inflammation, such as macrocalcifications, hyperemia, and the increase of parenchymal peak systolic velocity, suggesting that DNA fragmentation in this sperm population largely originates downstream of the epididymis.^17^

Finally, US evaluation also correlates with the degree of semen viscosity. The prevalence of hyperviscosity in subfertile patients was estimated around 26.2% and correlates with worse sperm parameters, in particular sperm motility and inflammation.^18^ The progressive anatomical extension of inflammation assessed by US is associated with a proportional increase of the viscosity of the seminal fluid measured centipoise, confirming that prostate‐vesicular‐epididymitis has a higher detrimental effect on sperm parameters than prostate‐vesiculitis and the latter in turn more than prostatitis alone.^19^

## 3 MICROBIOLOGICAL ASPECTS

The presence of prostate microcalcifications, macrocalcifications, and signs indicative of acinar ectasia (hypoechoic areas), and the occurrence of polycyclic endoluminal areas in the seminal vesicles represents US criteria associated with the persistence of bacteriospermia.^20^ Indeed, in these patients, TRUS allows identifying two different ultrasound patterns suggestive of chronic prostate‐vesiculitis that cannot be recognized with clinical history and physical examination alone. These US abnormalities reminiscent of prostate‐vesiculitis with or without micro‐abscess and prostate‐vesiculitis with ejaculatory duct sub‐obstruction found in patients with persistent infection are the clinical expression of chronic prostate‐vesiculitis. These two patterns also correlate with different sperm outcome, suggesting a role for TRUS in the follow‐up of chronic microbial MAGI.^20^ Furthermore, calcifications are associated with more severe forms of CP/CPPS since they can be found in ⁓47% of patients with CP/CPPS and associates with a higher rate of positivity to microbiological tests and lower rate of resolution of symptoms than in patients who do not have calcifications.^21^

US evaluation can also be useful in predicting the response to treatment. Indeed, after pharmacological treatment with levofloxacin, the first choice antibiotic to be used in patients with microbial MAGI, the percentage of eradication is significantly lower in patients with prostate‐vesiculo‐epididymitis compared to prostate‐vesiculitis or prostatitis alone. Moreover, bilateral prostate‐vesiculo‐epididymitis is associated with a lower rate of eradication after pharmacological treatment compared to unilateral prostate‐vesiculo‐epididymitis.^22^ Furthermore, in patients with prostate‐vesiculo‐epididymitis, sperm parameter abnormalities, increased ROS production and leukocytospermia persist even after three antibiotic courses, reinforcing the need for more aggressive treatments in these patients.^23^

HPV is another microorganism responsible for infertility and MAGI. In fact, HPV‐DNA can be found in the spermatozoa of 2–31% of the general male population and 10–35% of men undergoing assisted reproductive technique (ART). In a previous study, we found a prevalence of HPV in 20.8% of patients with inflammatory MAGI and 28.8% of microbial MAGI compared to 10% of controls suggesting that viral DNA testing should be done in these patients.^24^ Patients with MAGI and HPV infection have also different US patterns than their counterparts with Chlamydia trachomatis infection or amicrobial MAGI. Indeed, patients with HPV show a total number of US criteria indicative of MAGI significantly higher compared to the other two groups. Moreover, the infection with HPV is more frequently associated with complicated forms (bilateral prostate‐vesiculo‐epididymitis) and FSUF variant compared to the other groups. Finally, patients with HPV infection show a higher number of US criteria for prostatitis concentrated in the periurethral and transitional region of the prostate compared with the other groups. Accordingly, US evaluation represents a diagnostic element that helps to confirm this diagnostic hypothesis.^25^ Furthermore, the persistence of HPV in the seminal fluid of patients with MAGI, in particular for infections with oncogenic genotypes, also correlates with the persistence of US signs of inflammation.^26^

**FIGURE 1:**
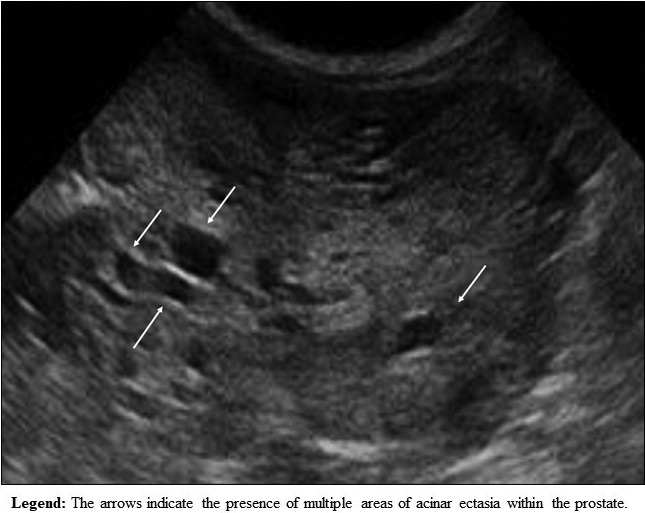
Areas of acinar ectasia within the prostate parenchyma

**FIGURE 2:**
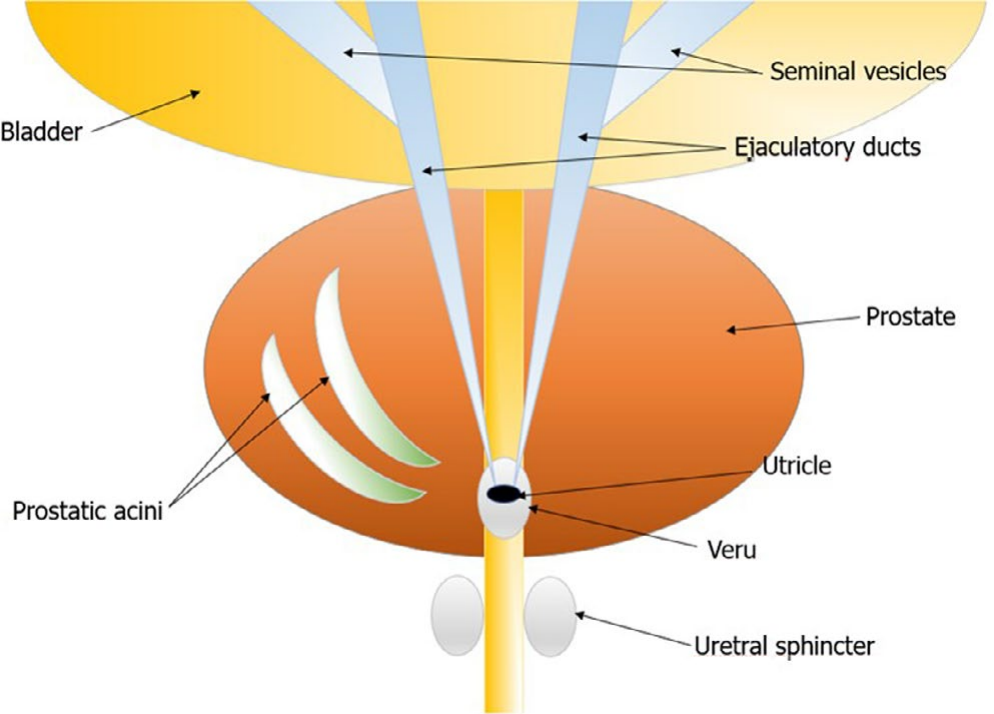
Sketch of the areas of acinar ectasia within the prostate parenchyma

**FIGURE 3:**
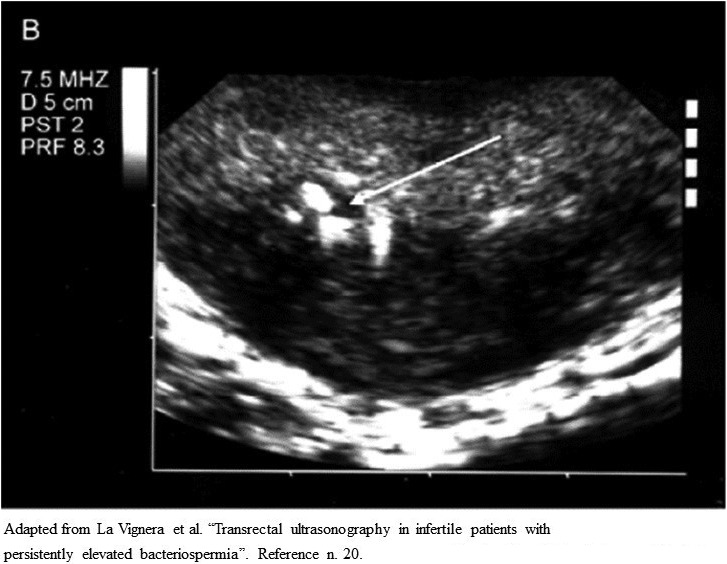
Ultrasound imaging of prostate in patients with persistent bacteriospermia

TRUS could also improve the specificity of the bacteriological examination performed on prostatic secretion obtained after massage (US integrated Meares and Stamey test).^27^ Indeed, thanks to US guidance, the physician could perform the massage on the parenchymal areas with acinar ectasia (Figures 1 and 2) and/or microcalcifications/macrocalcifications (Figure 3) that are often the sites hosting microbial agents.^28^

**FIGURE 4:**
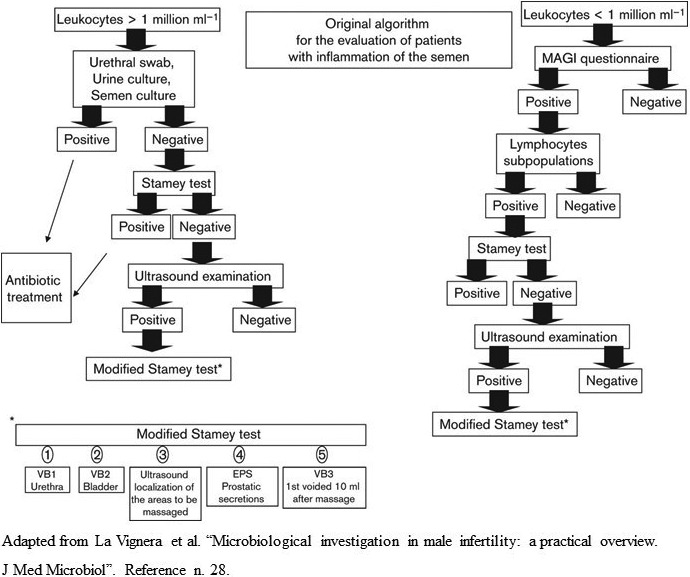
Diagnostic algorithm for patients with and without leukocytospermia

Finally, the microbiological evaluation is required for patients with leukocytospermia (leukocyte concentration >1 million/ml).^29^ However, the presence of US criteria suggestive for persistent inflammation of the prostate and seminal vesicles might suggest integrating leukocyte assessment with the use of monoclonal antibodies, such as CD45 for the characterization of other lymphocyte subpopulations. This in turn is related to the chronicity of the inflammatory process and the consequent transformation from the functional neutrophilic response to the lymphocytic one.^28^ From this point of view, a different algorithm can be hypothesized in clinical practice in consideration of the presence or absence of leukocytospermia in patients with US signs of prostatic inflammation (Figure 4).^28^ In particular, patients without leukocytospermia but with ultrasound signs of prostate inflammation should integrate the Mares and Stamey test with TRUS.

## 4 ENDOCRINOLOGICAL ASPECTS

**FIGURE 5:**
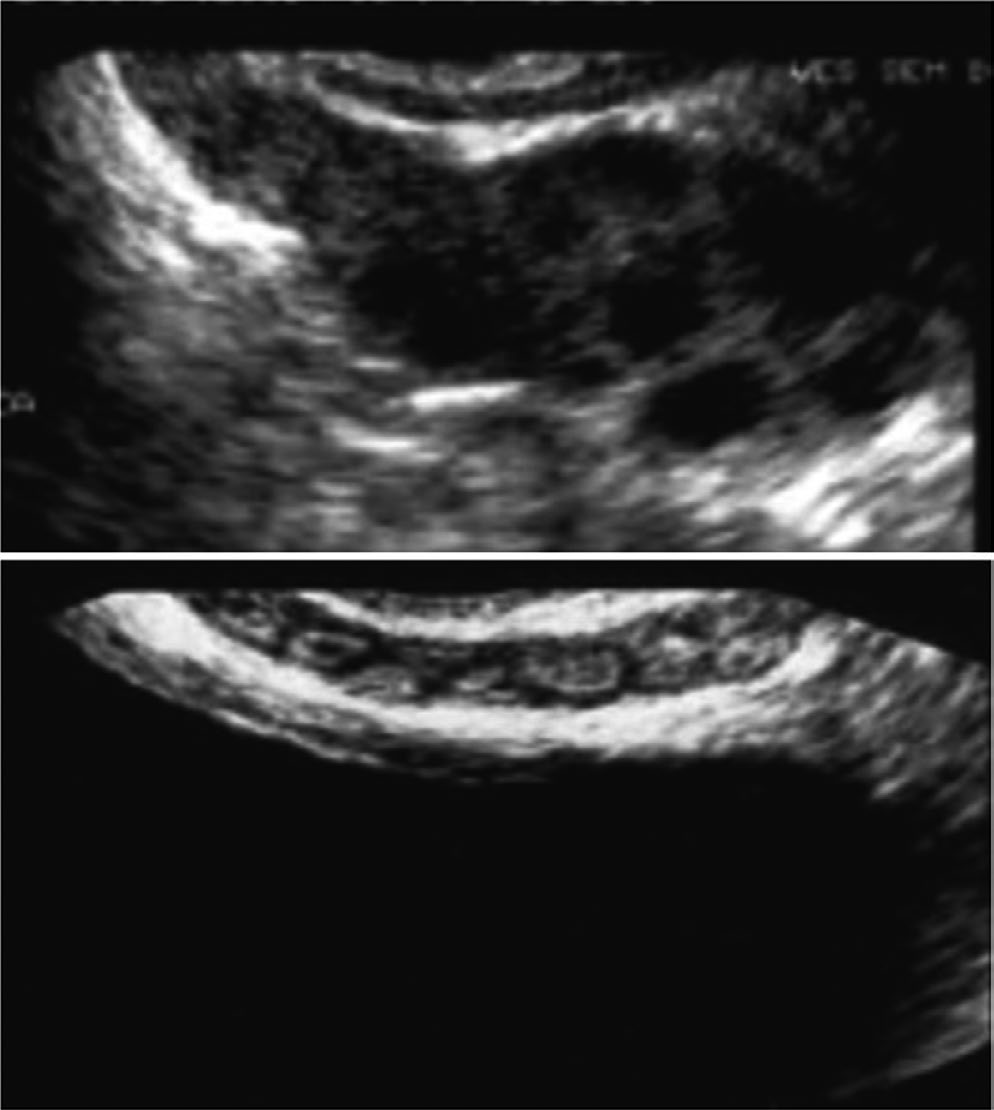
Dilated seminal vesicle compared with a seminal vesicle with reduced thickness^10^

In the experimental model, hypogonadism represents a risk factor for the progression of prostatic inflammation.^30^ However also in clinical practice, lower concentrations of total testosterone are associated with a higher frequency of prostatitis‐like symptoms and, in particular, with severe LUTS, such as reduced maximal flow rate (<10 ml/s) and higher post‐void residual urine volume (at least 100 ml).^31^ This is probably due to the anti‐inflammatory effects of androgens. Indeed, hypogonadism increases inflammatory markers (TNFα and IL‐6). Conversely, testosterone replacement therapy in patients with hypogonadism decreases inflammatory markers (C‐reactive protein, TNFα, and IL‐1).^31^ From the US point of view, we found a correlation between low serum total testosterone levels and higher frequency of complicated forms of MAGI (bilateral prostate‐vesiculo‐epididymitis and prostate‐vesiculitis), confirming the protective role of androgens against a greater extension of the inflammatory process. This suggests using TRUS in predicting patients who should undergo blood sampling for testosterone measurements.^32^ Moreover, eugonadal men have a lower prevalence of the FSUF variant of MAGI that, as above mentioned, represents a US form associated with persistent low‐quality sperm parameters after pharmacological treatment ^11^, ^32^ (Figure 5).

Another endocrinological condition often related to MAGI is type 2 diabetes mellitus (DM2). We have previously reported that MAGI is present in 43% of patients with DM2^33^ with a higher prevalence in DM2 patients with autonomic neuropathy compared to those without this complication.^9^ Indeed, these patients present peculiar US features involving the seminal vesicles. In particular, there are minimal or no changes in the anteroposterior diameter of the seminal vesicles after ejaculation and the altered anatomical ratio between the proximal and distal portion of the glandular body. The presence of these US characteristics suggests the possible use of TRUS in the early diagnosis of DM2 complicated with autonomic neuropathy.^9^, ^34^, ^35^, ^36^ Furthermore, the administration of selective inhibitors of V phosphodiesterase, commonly used for the treatment of erectile dysfunction, is associated with US modifications of the prostate‐vesicular tract in DM2 patients. These include increased differences in the anteroposterior diameter of the seminal vesicles before and after ejaculation and ejaculation fraction of the seminal vesicles. US changes also correlate with increased seminal fluid concentration of fructose and sperm parameter improvement. These findings suggest the use of PDE5i as a valid therapeutic strategy to treat the consequences of diabetic neuropathy on male reproductive health.^37^

## 5 SYSTEMIC ASPECTS

Prostatitis is often associated with irritable bowel disease. In a previous study, we found a prevalence of this disease in 30.3% of patients with prostatitis and 31.9% of prostatitis in patients with irritable bowel disease.^38^ In particular, patients with chronic bacterial prostatitis associated with irritable bowel disease have a higher frequency of MAGI compared with patients with chronic bacterial prostatitis without irritable bowel disease.^39^ Furthermore, the contemporary presence of both conditions is associated with greater severity of prostatic symptoms.^38^ Probably, the same mechanism involved in the pathogenesis of irritable bowel disease symptoms, such as an imbalance between commensal and pathogen bacteria of the intestinal microflora, local low‐grade inflammation associated with abnormal immune function, altered intestinal motility, and the intraluminal environment may play a role in the chronicization of bacterial prostatitis.^38^ In these patients, the main US characteristic indicative of MAGI is represented by the dilation of the periprostatic venous plexus.^40^ This finding could represent another mechanism by which rectal inflammation can affect the male accessory glands, given also the anatomical proximity of these structures.^40^

## 6 UROLOGICAL ASPECTS

Patients with varicocele, frequently have a concomitant dilation of the periprostatic venous plexus.^41^ In a previous study, we reported that patients with concomitant presence of these two venous alterations, maintain seminal fluid hyperviscosity and consequently decreased sperm motility after surgical varicocele repair compared with patients without dilation of the periprostatic venous plexus. This suggests lower effectiveness of varicocele correction on sperm parameters in these patients.^42^ In this study, we excluded MAGI as a confounding factor; however, in clinical practice, MAGI may associate with the presence of varicocele. In these cases, an adequate diagnostic path, also based on a US approach, allows the clinician to be guided toward the most appropriate therapeutic decision.

A recent study also showed an increased risk of prostate cancer in patients with MAGI and a positive correlation of this condition with the Gleason score; thus suggesting that the presence of chronic inflammation may be associated with more aggressive forms of cancer. In particular, the prevalence of prostate cancer was significantly higher in patients with US signs of prostatitis alone and prostate‐vesiculitis.^43^ However, this aspect still needs to be further investigated.

## 7 SEXUAL ASPECTS

We have previously shown a higher prevalence of sexual dysfunction in patients with MAGI. In particular, this was more frequent in patients who, in addition to the diagnosis of MAGI made according to the WHO criteria, present also typical US signs, suggesting once again the importance of US for a better characterization of MAGI.^44^ The presence of sexual dysfunctions in these patients is probably to be associated with the neuropathy resulting from chronic prostatic and periprostatic inflammation given the proximity of these structures to the nerve pathways responsible for the erectile and ejaculatory mechanisms.^44^ According to our findings, Screponi and colleagues found a prevalence of 62.1% of premature ejaculation in patients with CP.^45^ Similarly, a Chinese study found a higher prevalence of premature ejaculation and erectile dysfunction in patients with CP and the duration of the chronic inflammatory process.^46^ Finally, a meta‐analysis showed an overall prevalence of 62% of sexual dysfunctions in patients with CP/CPPS. In particular, the prevalence of erectile dysfunction and premature ejaculation was 29% and 40%, respectively.^47^

At a US level, the presence of acquired premature ejaculation in patients with MAGI correlates with a significant increase in the anteroposterior diameter of the caudal tract of the epididymis and seminal vesicles. In turn, these parameters showed a positive correlation with scores of the Premature Ejaculation Diagnostic Tool.^48^

## 8 CONCLUSIONS

US evaluation of the epididymal and the prostate‐vesicular tract in patients with MAGI is important for the following aspects:

1. Evaluation of the anatomical site of inflammation;
2. To diagnose between unilateral or bilateral forms;
3. Prognostic evaluation before pharmacological treatment;
4. Evaluation of the possible persistence after pharmacological treatment;
5. Prognostic evaluation of the outcome on sperm parameters;
6. Additional criteria for differential diagnosis of MAGI associated with HPV;
7. Early identification of any concomitant testosterone deficiency and complications of diabetes mellitus;
8. Early identification of patients with concomitant irritable bowel disease;
9. A better evaluation of patients undergoing varicocelectomy;
10. Assessment of the possible risk of developing prostate cancer;
11. A better evaluation of patients with MAGI‐related sexual dysfunction;
12. Integrate the microbiological diagnosis (eg Stamey Test) for a better definition of the clinical condition.

**FIGURE 6:**
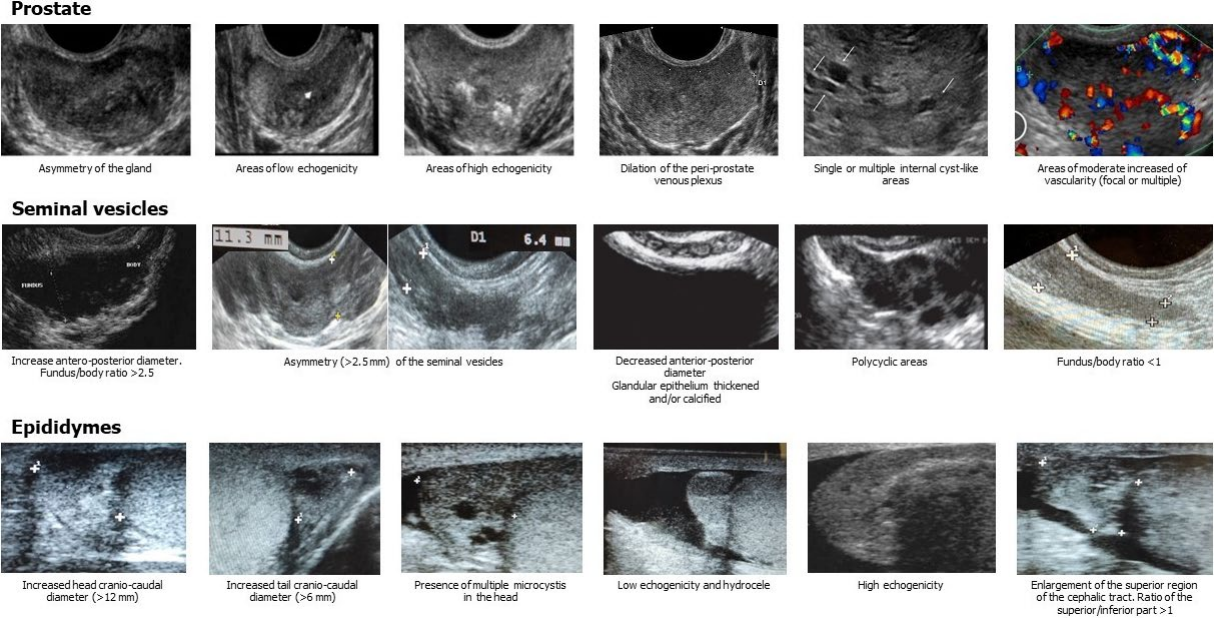
MAGI Diagnostic Criteria: Ultrasound Panel

Figure 6 illustrates in a single panel the suggestive ultrasound criteria for MAGI detectable in clinical practice.

## CONFLICT OF INTEREST

All authors declare no competing interests.

## AUTHORS’ CONTRIBUTIONS

Conceptualization: A.C. and S.L.V.; writing original draft preparation: S.L.V and A.C.; writing, review and editing: A.E.C.; R.A.C; visualization: R.C.; Data curation: L.M.M., R.C and F.B.; supervision: R.A.C.; project administration: A.E.C. and S.L.V. All authors have read and agreed to the published version of the manuscript.
